# A Double-Blinded Randomized Study Investigating a Possible Anti-Inflammatory Effect of Saxagliptin versus Placebo as Add-On Therapy in Patients with Both Type 2 Diabetes And Stable Coronary Artery Disease

**DOI:** 10.1155/2017/5380638

**Published:** 2017-05-15

**Authors:** Ida Unhammer Njerve, Sissel Åkra, Thomas W. Weiss, Svein Solheim, Reidun Øvstebø, Hans Christian D. Aass, Rune Byrkjeland, Harald Arnesen, Ingebjørg Seljeflot

**Affiliations:** ^1^Center for Clinical Heart Research, Department of Cardiology, Oslo University Hospital Ullevaal, Oslo, Norway; ^2^Center for Heart Failure Research, Oslo University Hospital Ullevaal, Oslo, Norway; ^3^Faculty of Medicine, University of Oslo, Oslo, Norway; ^4^3rd Medical Department for Cardiology, Wilhelminenspital, Vienna, Austria; ^5^The Blood Cell Research Group, Department of Medical Biochemistry, Oslo University Hospital Ullevaal, Oslo, Norway

## Abstract

**Background:**

Promising results regarding potential anti-inflammatory and antiatherosclerotic effects of gliptins have been reported. Our aim was to investigate whether saxagliptin treatment modifies expression of inflammatory markers, primarily in peripheral blood mononuclear cells (PBMCs) and in circulating leukocytes in patients with stable coronary artery disease (CAD) and T2DM.

**Methods:**

Patients (*n* = 12) were randomized to saxagliptin 5 mg daily or placebo for 3 months. Samples were taken at baseline and end of study in fasting state prior to intake of medications. PBMCs were isolated and cryopreserved at −150°C until ex vivo exposed to 1 ng/mL of lipopolysaccharide (LPS) for 4 hours. Gene expression was performed with custom-designed TaqMan® Arrays and relative quantification by real-time PCR (RT-qPCR).

**Results:**

HbA1c was reduced in the saxagliptin-treated group compared to that in the change with placebo (*p* = 0.042). In unstimulated PBMCs and in circulating leukocytes, we observed a significant increase in IL-10 expression in the saxagliptin group (*p* = 0.043, both), significantly different from that in the placebo (*p* = 0.009 and *p* = 0.032, resp.). No between group differences in changes were observed in any of the selected proinflammatory markers.

**Conclusion:**

In our small cohort of patients with combined T2DM and CAD, a possible anti-inflammatory effect of saxagliptin, observed in the present study by upregulation of IL-10 in leukocytes, needs to be confirmed in larger studies.

## 1. Background

Gliptins or dipeptidyl peptidase-4 (DPP-4) inhibitors are used as either monotherapy or, more frequently, as add-on therapy to other oral antidiabetic drugs in type 2 diabetes (T2DM) [1, 2]. They delay the inactivation of incretins glucagon-like peptide-1 (GLP-1) and glucose-dependent insulinotropic polypeptide (GIP) after meals, reducing postprandial glucose levels and also HbA1c levels [3].

DPP-4 is an enzyme with substrates other than the incretin hormones, including cytokines that might influence inflammation and atherosclerosis [4]. Animal studies have shown promising results regarding anti-inflammatory and antiatherosclerotic effects of gliptins in general [5–7]. Also, some clinical studies have been published showing a potential effect on atherosclerosis and inflammation in patients with T2DM using DPP-4 inhibitors as compared to that in patients using other antidiabetic drugs or placebo [8–11]. This has led to the hypothesis that DPP-4 inhibitors might have beneficial anti-inflammatory effects in addition to glucose-lowering effects.

The aim of our study was therefore to investigate whether the DPP-4 inhibitor saxagliptin modifies the expression of selected inflammatory biomarkers, primarily in isolated peripheral blood mononuclear cells (PBMCs) and in circulating leukocytes in patients with stable coronary artery disease (CAD) and T2DM. In addition, circulating levels of the markers were measured.

The selected markers were tumor necrosis factor-*α* (TNF-*α*), interleukin-1*β* (IL-1*β*), IL-18, monocyte chemoattractant protein-1 (MCP-1), fractalkine (CX3CL1) and its receptor CX3CR1, and the anti-inflammatory cytokine IL-10.

## 2. Materials and Methods

### 2.1. Patients and Study Design

Patients (*n* = 12) with both T2DM and stable CAD recruited from the Department of Cardiology, Oslo University Hospital Ullevaal, Oslo, Norway, from 2012–14, were randomized double blinded to placebo or saxagliptin (5 mg/day) for 3 months. All study participants were adults, had angiographically verified stable CAD, and had a history of T2DM. Inclusion criteria were use of metformin and/or glimepiride for their T2DM and HbA1c levels >6.5%. Exclusion criteria for the study included allergy or hypersensitivity to any of the drug's components, heart failure NYHA class III and IV, severe liver failure, moderate or severe kidney failure, malignant disease, active infectious disease, acute coronary syndrome during the last three months, pregnancy, or breastfeeding.

All patients gave written, informed consent to participate in the study. The study was conducted in accordance with the Declaration of Helsinki and consistent with Good Clinical Practice, and the Regional Ethics Committee and the Norwegian Medicines Agency approved the protocol. ClinicalTrials.gov identifier: NCT01552018.

### 2.2. Sampling Procedures

Blood samples, including PAX gene blood RNA tubes (PreAnalytix Qiagen GmBH, Germany) and BD vacutainer CPT tubes for isolation of PBMC (Becton, Dickinson and Company, Franklin Lakes, USA), were drawn by standard venipuncture between 08.00 and 10.00 am after an overnight fast and before intake of morning medication at inclusion and after 3-months of follow-up. Serum and plasma for determination of selected biomarkers were prepared by centrifugation within 1 hour at 2500 ×g for 10 min and 4°C at 3000 ×g for 20 min, respectively. The samples were stored at −80°C until analyses.

PBMCs were isolated and cryopreserved within 2 hours. In brief, cells were isolated by centrifugation at several steps, added 5% FCS/RPMI, and counted for the distribution of monocytes and lymphocytes, recorded by flow cytometry using BD Accuri C6 (BD Biosciences, 2350 Qume Drive, San Jose, CA). Diluted DMSO/RPMI (20%) was added before freezing at −150°C. Cells were further ex vivo exposed to lipopolysaccharide (LPS) with a final concentration of 1 ng/mL for 4 hours. Equal number of cells was used in all experiments. The method is according to what is previously reported with a minor modification [12].

### 2.3. Laboratory Analyses

Fasting glucose, HbA1c, C-peptide, and insulin were determined by conventional laboratory methods. Glucose was analyzed by enzyme immunoassay (Cobas 8000, Roche Diagnostics, Basel, Switzerland), HbA_1c_ by turbidimetric inhibition immunoassay (Roche Diagnostics), insulin by DELFIA method (Perkin Elmer, Waltham, Massachusetts, USA), and C-peptide by electrochemiluminescence immunoassay (ECLIA) (Roche Diagnostics).

For gene expression analyses, total RNA was isolated from unstimulated and stimulated PBMCs by use of the RNeasy® Mini Kit (Qiagen) and from PAXgene tubes by use of a PAXGENE® Blood RNA Kit (PreAnalytix, Qiagen, GmBH), with an extra cleaning step (RNeasy®MinElute® Cleanup Kit, Qiagen). RNA concentration (ng/*μ*L) and quality was measured by the NanoDrop™ 1000 Spectrophotometer (Thermo Scientific, Wilmington, Delaware, USA).

A predefined RNA concentration of 5 ng/*μ*L was used for cDNA synthesis performed with qScript™cDNA superMix (Quanta Biosciences, Gaithersburg, Maryland, USA).

Real-time PCR was performed on ViiA™ 7 with TaqMan custom-designed arrays for expression in both the circulating whole blood and PBMCs (Applied Biosystems, Foster City, CA, USA). The gene expression assays for TNF-*α*, IL-1*β*, IL-18, MCP-1, CX3CL1, and CX3CR1 were Hs01113624_g1, Hs01555410_m1, Hs01038788_m1, Hs00234140_m1, Hs 00171086_m1, and Hs 01922583_s1, respectively. All formats used *β*2-microglobulin (HS99999907_m1) as endogenous control and TaqMan Universal PCR Master Mix (P/N 4324018). mRNA levels were determined by relative quantification (RQ) using the ΔΔCT method [13].

Levels of biomarkers in the PBMC supernatants were assessed by customized magnetic Luminex Screening Assays multiplex kit (R&D) with the Bio-plex® luminex xMAP™ technology. Circulating markers were determined by commercially available enzyme-linked immunosorbent assay (ELISA) kits from R&D Systems (Abingdon, Oxon, UK).

### 2.4. Statistics

Calculations were performed using SPSS version 22.0 (SPSS Inc., USA). *p* values <0.05 were defined as statistically significant. Data are given as proportions or median (25, 75 percentiles). Nonparametric statistics were used due to small sample size and several skewed variables. Differences between groups and differences in changes between the groups were analyzed by Mann–Whitney *U* test. Fisher's exact 2-sided test was used for categorical variables. Within group changes were calculated with Wilcoxon signed-ranks test.

## 3. Results

Baseline characteristics of the patients, including use of medications, are given in Table 1. One patient in the saxagliptin group was excluded from analyses at the last visit due to elevated C-reactive protein (CRP) (>10 mg/L).

Two patients in the saxagliptin group experienced adverse events during the study period—one patient had urinary tract infection and another an episode of mild hypoglycemia.

HbA1c was significantly reduced in the saxagliptin-treated group (*p* = 0.042), different from that in the placebo group (*p* = 0.017) (Table 2). No other significant changes or differences in changes were observed between the groups for other glucometabolic variables (Table 2).

### 3.1. Gene Expression in Isolated PBMCs

The mean distribution of monocytes and lymphocytes in the mononuclear cells from all experiments was 9.6% and 90.4%, respectively.

Levels of gene expression of the inflammatory markers in unstimulated and LPS-stimulated cells are shown in Table 3. Expected increase in LPS-stimulated cells were seen, except for MCP-1, which might be due to the short incubation time [14], and also for CX3CR1.

**Figure 1:**
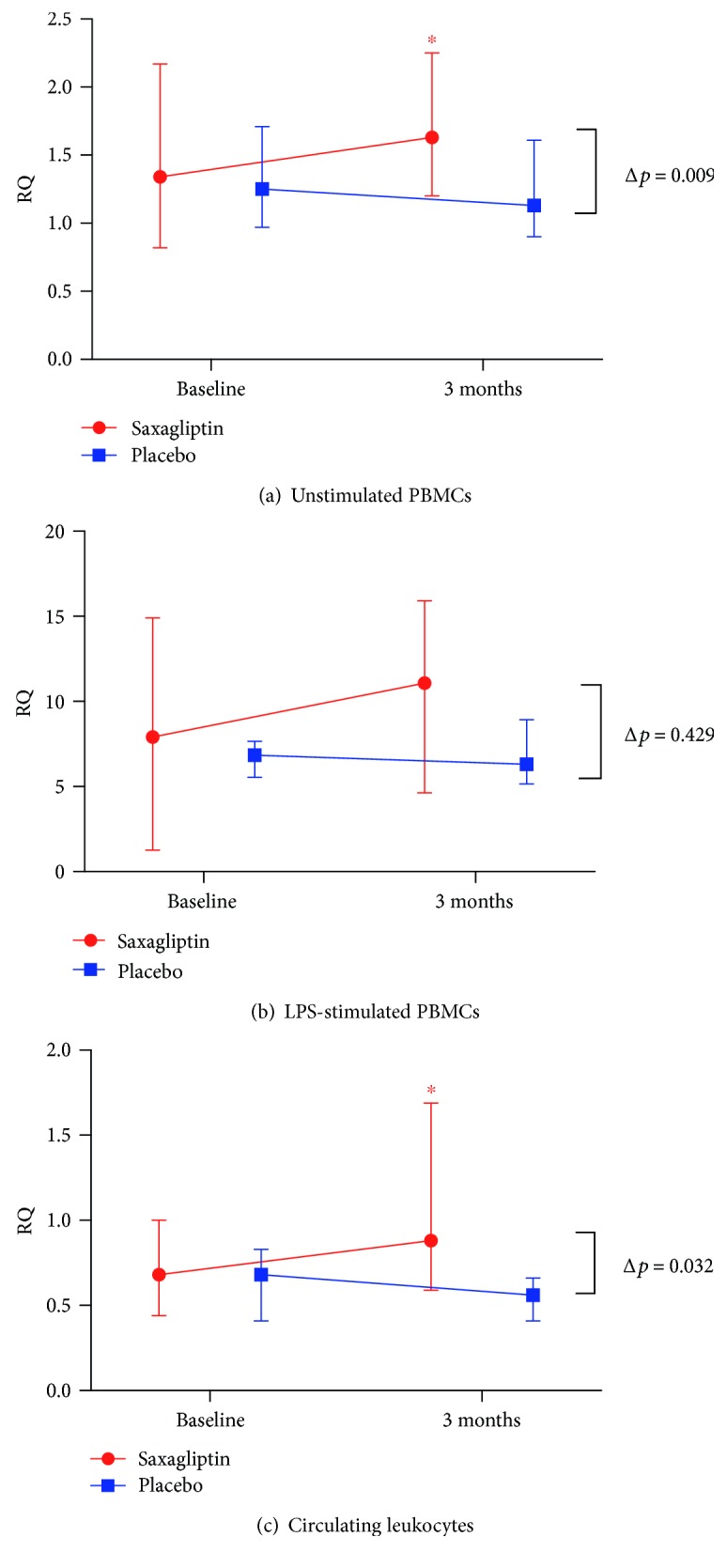
The figure shows RQ values of IL-10 from (a) unstimulated PBMCs, (b) LPS-stimulated PBMCs, and (c) circulating leukocytes at baseline and after 3 months in the two randomized groups. The median values are given, with 25 and 75 percentiles depicting the spread. Δ*p* denotes relative differences in change between the groups. ^∗^ depicts significant intragroup change in RQ value.

In unstimulated cells, we observed a significant increase in IL-10 expression from baseline in the saxagliptin-treated patients (*p* = 0.043), significantly different from that in the placebo (*p* = 0.009), however not in LPS-stimulated cells. No differences in changes between the groups in the expression of the proinflammatory markers were observed, neither in unstimulated nor in LPS-stimulated cells (Table 3, Figures 1(a) and 1(b)).

### 3.2. Supernatant from Isolated PBMCs

No between group differences or differences in changes of the selected markers were recorded in supernatants from neither unstimulated nor LPS-stimulated cells (data not shown). IL-18 was not detectable in unstimulated cells with the assay used.

### 3.3. Gene Expression in Circulating Leukocytes

**Figure 2:**
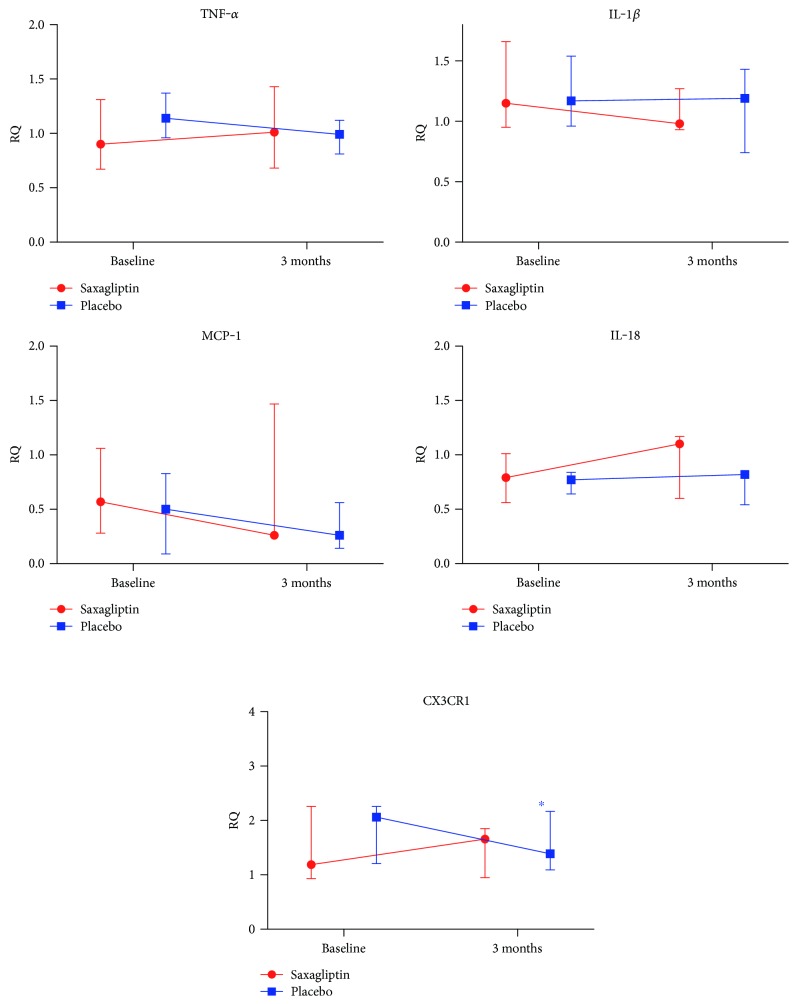
mRNA RQ values in circulating leukocytes before (baseline) and after treatment (3 months). Median values are given, with 25 and 75 percentiles depicting the spread. ^∗^Intragroup change, *p* = 0.043. There were no statistically significant differences in relative changes of the selected proinflammatory markers between the groups.

There were no significant differences between the groups in gene expression of the selected markers at baseline. IL-10 mRNA increased significantly after 3 months in the saxagliptin group (*p* = 0.043), significantly different from that in the controls (*p* = 0.032) (Figure 1(c)). There was an intragroup reduction in CX3CR1 expression in the placebo group (*p* = 0.043), however not significantly different from the change in the saxagliptin group (*p* = 0.151) (Figure 2).

### 3.4. Circulating Levels

There were no statistically significant differences in the circulating levels between the groups at baseline. IL-18 levels increased numerically in both groups during the intervention period, however, only significant in the saxagliptin treatment group (*p* = 0.043) (different from placebo, *p* = 0.009) (Table 4). IL-10 levels were all below detection limit of our assay.

## 4. Discussion

In a recent review, attempting to compare different DPP-4 inhibitor effects on pro- and antiatherogenic factors, only few studies on saxagliptin have been performed [15].

Saxagliptin treatment did not reduce the LPS induced proinflammatory response in PBMCs in our study. We could also not demonstrate any downregulation of proinflammatory genes or reduced proinflammatory circulating markers related to saxagliptin treatment. However, in the present study, an increase in IL-10 mRNA expression in isolated PBMCs was demonstrated, which is in line with previous results on sitagliptin [10]. The increase in IL-10 expression shown in circulating leukocytes strengthens our result. From the literature, IL-10 is primarily produced from subsets of T cells, macrophages, and dendritic cells [16]. However, which cell types that are involved in the upregulation seen in our study can only be speculated on, but lymphocytes, which are dominantly present in PBMCs and also highly present among circulating cells, might be the main source. We could, however, not find a corresponding increase in supernatants of the cell cultures, which might be due to the experiment time frame [17], and levels of IL-10 in the circulation were also not detectable, although a high sensitivity assay was used.

The increase in IL-10 levels might be speculated upon as a possible anti-inflammatory effect of saxagliptin. However, it might also be related to the observed reduction in HbA1c, which fits with studies showing a reduction of IL-10 in response to elevated glucose levels [18, 19].

We observed an increase in circulating IL-18 in the patients treated with saxagliptin. This is in contrast to another study reporting a significant reduction in IL-18 with gliptins [20]. This report was, however, not from patients with cardiovascular disease, and two other DPP-4 inhibitors were used. It should, nevertheless, be emphasized that the number of patients was low and the values highly spread in our study, and the results should therefore be interpreted with caution. In another study, it was reported on no effect of long-term treatment (24 months) with alogliptin on other selected inflammatory markers [11].

All patients included in our study were using medications also for their CAD; thus, all were using statins and many antiplatelet therapies. Therefore, a possible anti-inflammatory effect might be masked by these drugs, which also have anti-inflammatory effects [21, 22]. The use of medications in general in such populations, may complicate the reliability of the results. Nevertheless, this is the normal situation in such patients and new antidiabetic drugs might add to the positive effects of other drugs.

The large SAVOR-TIMI 53 study showed that adding saxagliptin to standard of care did not increase nor decrease cardiovascular ischemic events in high-risk patients. But they did, however, show a 27% greater risk of hospitalization for heart failure [23]. Also, other clinical trials have been neutral with regard to DPP-4 inhibitor treatment on cardiovascular event rates [24, 25]. The conclusion so far has been that the investigated DPP-4 inhibitors have been noninferior for cardiovascular safety, however not demonstrating superiority, meaning beneficial effects of DPP-4 inhibition on cardiovascular risk [26]. A new drug, empagliflozin (sodium-glucose cotransporter 2 (SGLT2) inhibitor), has recently gained a lot of interest since it seems to reduce cardiovascular mortality compared to placebo [27].

The main limitation of our study is the low number of patients included. Thus, this is a small, purely hypothesis generating study, with potential type 2 statistical error, and our results need to be discussed and interpreted with caution. Nevertheless, for the ex vivo model of isolated PBMCs, the number might be considered satisfactory. We also obtained expected reductions in HbA1c levels in the saxagliptin group, indicative of good compliance to the study drug.

## 5. Conclusion

Taken together, saxagliptin treatment in patients with combined CAD and T2DM for 3 months did not downregulate proinflammatory markers in neither isolated PBMCs nor circulating leukocytes, whereas the anti-inflammatory cytokine IL-10 was upregulated in both models. A possible anti-inflammatory effect of saxagliptin, as observed in the present study with increased expression of IL-10, needs to be confirmed in larger studies.

## Figures and Tables

**Table 1:** Baseline characteristics according to the randomized groups. Median (25, 75 percentiles) or number is given.

Characteristics	Saxagliptin	Placebo
Age (years)	68.5 (63.0, 73.0)	64.0 (58.5, 67.5)
Number (female)	6 (0)	6 (1)
Years of diabetes	7.8 ± 4.4	9.2 ± 3.6
Years of CAD	6 (1, 15)	2 (1, 7)
Hypertension (*n*)	5	6
Previous AMI (*n*)	3	3
Current smoker (*n*)	2	1
SBP (mmHg)	128 (121, 144)	133 (123, 145)
DBP (mmHg)	75 (68, 79)	74 (69, 80)
Waist circumference (cm)	103 (90, 117)	105 (94, 110)
BMI (kg/m^2^)	28.1 (25.1, 32.3)	28.6 (24.7, 29.0)

Medication (*n*)		

Glimepiride	2	1
Metformin	5	6
Platelet inhibitors	4	5
ACE-inhibitors or ARBs	4	6
Betablocker	5	6
Statin	6	6
Diuretic	3	1

AMI: acute myocardial infarction; SBP: systolic blood pressure; DBP: diastolic blood pressure; BMI: body mass index; ACE: angiotensin converting enzyme; ARB: angiotensin II receptor blocker.

**Table 2:** Glucometabolic variables according to the randomized groups at baseline and after 3 months. Median (25, 75 percentiles) is given.

Parameter	Baseline		3 months		Δ*p*
	Saxagliptin (*n* = 6)	Placebo (*n* = 6)	Saxagliptin (*n* = 5)	Placebo (*n* = 6)	
HbA1c (%)	7.5 (7.1, 8.3)	7.3 (6.8, 7.7)	6.9 (6.7, 7.4)^∗^	7.2 (6.9, 7.7)	**0.017**
Glucose (mmol/L)	8.2 (7.7, 9.7)	8.5 (7.4, 10.6)	7.2 (6.5, 10.0)	8.5 (7.7, 10.4)	0.329
Insulin (pmol/L)	72 (55, 139)	60 (43, 104)	58 (52, 116)	71 (38, 119)	0.818
C-peptide (pmol/L)	1090 (810, 1573)	1025 (767, 1345)	897 (758, 1599)	887 (744, 1649)	0.818

^∗^ refers to intragroup change in the saxagliptin group, *p* = 0.042; Δ*p* refers to difference in changes from baseline to 3 months between the groups.

**Table 3:** mRNA RQ values of the selected markers in unstimulated and LPS-stimulated PBMCs before and after treatment in the randomized groups. Median (25, 75 percentiles are given).

Unstimulated						LPS stimulated				
	Baseline		3 months			Baseline		3 months		
	Saxagliptin (*n* = 6)	Placebo (*n* = 6)	Saxagliptin (*n* = 5)	Placebo (*n* = 6)	Δ*p*1	Saxagliptin (*n* = 6)	Placebo (*n* = 6)	Saxagliptin (*n* = 5)	Placebo (*n* = 6)	Δ*p*2
TNF-*α*	0.57 (0.44, 0.78)	0.47 (0.43, 0.76)	0.68 (0.56, 0.96)	0.42 (0.24, 0.85)	0.177	4.20 (2.99, 6.04)	3.60 (3.40, 4.31)	5.13 (4.14, 16.70)	4.29 (3.47, 4.96)	1.000
IL-1*β*	0.94 (0.73, 1.21)	0.67 (0.59, 1.10)	1.18 (0.84, 1.76)	0.58 (0.38, 0.96)	0.082	19.36 (8.99, 22.50)	11.11 (9.56, 13.65)	15.77 (11.15, 22.18)	14.08 (8.61, 16.47)	0.662
MCP-1	0.46 (0.30, 0.71)	0.47 (0.38, 0.73)	0.50 (0.38, 0.85)	0.45 (0.26, 0.73)	0.792	0.17 (0.04, 0.42)	0.15 (0.08, 0.22)	0.21 (0.07, 0.41)	0.16 (0.09, 0.27)	0.429
IL-18	1.22 (0.87, 1.49)	1.10 (0.82, 1.32)	1.24 (0.79, 1.87)	1.01 (0.69, 1.20)	1.000	1.99 (1.06, 2.95)	1.76 (1.32, 2.08)	1.59 (0.41, 3.69)	1.95 (1.29, 2.86)	0.429
CX3CR1	0.69 (0.53, 1.29)	1.37 (0.64, 2.53)	0.58 (0.49, 1.06)	1.39 (0.86, 2.09)	0.931	0.44 (0.36, 0.84)	0.69 (0.39, 1.23)	0.48 (0.29, 1.17)	0.65 (0.35, 0.77)	0.662
IL-10	1.34 (0.82, 2.17)	1.25 (0.97, 1.71)	1.63^∗^ (1.20, 2.25)	1.13 (0.90, 1.61)	**0.009**	7.90 (1.26, 14.90)	6.84 (5.54, 7.65)	11.07 (4.63, 15.90)	6.30 (5.15, 8.93)	0.429

^∗^ *p* = 0.043 intragroup change in the saxagliptin group (Wilcoxon signed-rank test). Δ*p*1 denotes relative differences in changes between groups in unstimulated cells (baseline levels taken into account). Δ*p*2 denotes relative differences in changes between groups in LPS-stimulated cells (baseline levels taken into account).

**Table 4:** Circulating levels of the selected markers before and after treatment in the randomized groups. Median (25, 75 percentiles) are given.

Marker	Baseline		3 months		
	Saxagliptin (*n* = 6)	Placebo (*n* = 6)	Saxagliptin (*n* = 5)	Placebo (*n* = 6)	Δ*p*
TNF-*α* (pg/mL)	1.61 (1.36, 1.96)	1.23 (1.04, 1.49)	1.54 (1.32, 1.64)	1.33 (1.16, 1.59)	0.429
MCP-1 (pg/mL)	117 (100, 149)	118 (94, 173)	114 (99, 116)	113 (88, 166)	0.792
IL-18 (pg/mL)	297 (192, 493)	399 (307, 500)	342 (177, 422)^∗^	419 (305, 522)	**0.009**
Fractalkine (pg/mL)	604 (535, 649)	520 (455, 628)	577 (451, 627)	504 (443, 622)	0.931

Δ*p* denotes difference in relative change between groups (Mann–Whitney *U* test); ^∗^*p* = 0.043 for intragroup change (Wilcoxon signed-rank test).

## Abbreviations

CAD: Coronary artery disease
CRP: C-reactive protein
CX3CL1: Fractalkine
CX3CR1: Fractalkine receptor
DPP-4: Dipeptidyl peptidase 4
IL: Interleukin
LPS: Lipopolysaccharide
MCP-1: Monocyte chemoattractant protein-1
PBMCs: Peripheral blood mononuclear cells
T2DM: Type 2 diabetes
TNF-*α*: Tumor necrosis factor alpha.
